# Prevalence and Antimicrobial Resistance of *Escherichia coli*, *Salmonella* and *Vibrio* Derived from Farm-Raised Red Hybrid Tilapia (*Oreochromis* spp.) and Asian Sea Bass (*Lates calcarifer*, Bloch 1970) on the West Coast of Peninsular Malaysia

**DOI:** 10.3390/antibiotics11020136

**Published:** 2022-01-20

**Authors:** Rita Rosmala Dewi, Latiffah Hassan, Hassan Mohammad Daud, Mohd. Fuad Matori, Fauziah Nordin, Nur Indah Ahmad, Zunita Zakaria

**Affiliations:** 1 Faculty of Veterinary Medicine, Universiti Putra Malaysia, Serdang 43400, Selangor, Malaysia; gs51419@student.upm.edu.my (R.R.D.); hassanmd@upm.edu.my (H.M.D.); fuma@upm.edu.my (M.F.M.); fauziah_upm@upm.edu.my (F.N.); nurindah@upm.edu.my (N.I.A.); zunita@upm.edu.my (Z.Z.); 2 Institute of Bioscience, Universiti Putra Malaysia, Serdang 43400, Selangor, Malaysia

**Keywords:** aquaculture, prevalence, antibiotic resistance, multidrug resistance, colistin, *E. coli*, *Salmonella* spp., *Vibrio* sp.

## Abstract

Antibiotics are widely used in intensive fish farming, which in turn increases the emergence of antimicrobial-resistant (AMR) bacteria in the aquatic environment. The current study investigates the prevalence and determines the antimicrobial susceptibility of *E. coli*, *Salmonella*, and *Vibrio* in farmed fishes on the west coast of Peninsular Malaysia. Over a period of 12 months, 32 aquaculture farms from the Malaysian states of Selangor, Negeri Sembilan, Melaka, and Perak were sampled. Both *E. coli* and *Salmonella* were highly resistant to erythromycin, ampicillin, tetracycline, and trimethoprim, while *Vibrio* was highly resistant to ampicillin and streptomycin. Resistance to the antibiotics listed as the highest priority and critically important for human therapy, such as colistin in *E. coli* (18.1%) and *Salmonella* (20%) in fish, is a growing public health concern. The multi-drug resistance (MDR) levels of *E. coli* and *Salmonella* in tilapia were 46.5% and 77.8%, respectively. Meanwhile, the MDR levels of *E. coli*, *Salmonella*, *V. parahaemolyticus*, *V. vulnificus* and *V. cholerae* in Asian seabass were 34%, 100%, 21.6%, 8.3% and 16.7%, respectively. Our findings provide much-needed information on AMR in aquaculture settings that can be used to tailor better strategies for the use of antibiotics in aquaculture production at the local and regional levels.

## 1. Introduction

The increasing demand for aquaculture products as a source of protein stimulates the propagation and expansion of aquaculture in many countries. Malaysia, together with other Southeast Asian countries, is a major producer of aquaculture products [1]. In 2016, the local freshwater and brackish water aquaculture contributed 103,348 metric tonnes valued at MYR 1,091,463 million (USD 257,694) and 304,039 metric tonnes valued at MYR 2,509,717 million (USD 592,543), respectively [2]. Tilapia is a major freshwater species constituting 46% of total freshwater aquaculture production, with the red hybrid (*Oreochromis* spp.) as the predominant variety cultured [3]. Meanwhile, marine finfish including Asian seabass (*Lates calcalifer*) contributed about 37.6% of aquaculture production in 2012 [4]. 

Despite the high nutritional quality that links fish consumption to positive health effects in humans, the aquaculture system is tremendously vulnerable to pollution and run-offs from anthropogenic sources which contaminate fish products with microbiological hazards such as *E. coli* and *Salmonella.* Intensive farming may also encourage the use of antibiotics to ensure the health of aquatic animals beyond therapeutic needs [5,6]. Aquaculture products contaminated with human pathogens have been documented in many countries, with *Salmonella* spp. and *Vibrio* sp. being the most common pathogens reported in seafood [7,8]. In addition, drug-resistant *E. coli*, *Salmonella* and pathogenic *Vibrio* have been reported to circulate in aquaculture settings and their products [9,10,11,12]. The increasing level of resistance and multi-drug resistance (MDR) among pathogens to commonly used antibiotics in medical and veterinary therapies poses a great challenge to the treatment of human and animal diseases [13,14]. Moreover, AMR in the aquaculture sector plays a significant role in the globalization of AMR [15] through aquatic ecosystem interconnections. 

The global strategy for AMR published by the World Health Organization [16] states that multisector collaboration between human health, animal health and agriculture (including the tripartite collaboration agreed by the FAO/WHO/OIE) is needed to decelerate the global emergence of AMR. Aquatic animal health is one of the major targeted sectors included in the strategic AMR program of the FAO to support the WHO-led global action plan [17]. In the Malaysian Strategic Action Plan for AMR 2017–2021 (MyAP–AMR) [18], AMR in the aquaculture system and its products is addressed as one component in Malaysia’s national action [2]. Therefore, there is an ongoing comprehensive monitoring project for veterinary residues from aquaculture farms, even though AMR monitoring in fisheries is relatively in its infancy [18].

Sporadic studies on the AMR of bacteria isolated from the local fish and fish products are available [9,19,20]. These studies suggested that public-health-significant bacteria circulating fish farms are resistant toward a wide range of antibiotics. Hence, we hypothesized that *E. coli*, *Salmonella* sp. and *Vibrio* sp. isolated from tilapia and Asian seabass fish in the west coast of Peninsular Malaysia are highly resistant to multiple antimicrobial agents.

The objective of this study is to describe the prevalence and distribution of antimicrobial resistance of *E. coli*, *Salmonella* and *Vibrio* in cultured tilapia and Asian seabass fish in four states on the west coast of Peninsular Malaysia, providing the much-needed information about bacteria of public health interest in aquaculture in line with the Malaysian AMR national action plan. We also compare the resistance pattern of isolates in this study to that of isolates from local livestock to give a more holistic one-health view about the AMR situation. 

## 2. Results

### 2.1. Farm Demography

The study involved 32 cultured fish farms (tilapia farms = 19 and Asian seabass farms = 13). The production of tilapia was mainly in earthen ponds (*n* = 17, 89%), although two farms (11%) practiced the floating cage system of farming in ex-mining pools and man-made reservoirs. The floating cage system was mainly adopted in the production of Asian seabass (*n* = 10, 77%), with three farms (23%) employing earthen ponds at river estuaries.

### 2.2. Prevalence of E. coli, Salmonella spp. and Vibrio sp.

Overall, the prevalence of *E. coli* and *Salmonella* in tilapia fish was 44.5% and 0.6%, respectively, while in tilapia pond water, the occurrence was 100% and 15.7%, respectively (Table 1). *V. cholerae* was found in neither tilapia fish nor tilapia pond water (Table 2). 

The prevalence of *E. coli* and *Salmonella* in Asian seabass fish was 5.3% and 0.4%, respectively. *E. coli* presence in Asian seabass water was 61.5%, while *Salmonella* was not detected in the pond water (Table 1). Of the 265 cultured Asian seabasses, 35.5%, 2.3% and 1.9% were, respectively, positive for *V. parahaemolyticus*, *V. vulnificus*, and *V. cholerae.* The detection of these three bacteria in Asian seabass pond water was 46.2%, 7.7% and 7.7%, respectively (Table 2).

*Salmonella* was analyzed further for two serotypes, namely *S. typhimurium* and *S. enteritidis*, by using PCR. Of the 10 *Salmonella* isolates, two (20%) were identified as *S. typhimurium* and none were identified as *S. enteritidis*; they were accordingly grouped as *Salmonella* spp.

### 2.3. Antibiotic Susceptibility According to Species of Bacteria from Farm-Raised Tilapia

**Figure 1:**
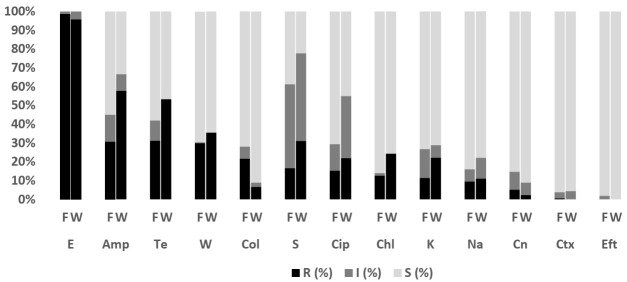
Antibiotic susceptibility pattern of *E. coli* isolates recovered from tilapia fish (*n* = 157) and pond water (*n* = 45) on the west coast of Peninsular Malaysia against antimicrobials tested. E, Erythromycin; Te, Tetracycline; Amp, Ampicillin; W, Trimethoprim; Col, Colistin; S, Streptomycin; Cip, Ciprofloxacin; Chl, Chloramphenicol; K, Kanamycin; Na, Nalidixic Acid; Cn, Gentamycin; Ctx, Cefotaxime; Eft, Ceftiofur. F: Fish; W: Pond Water; R: Resistant; I: Intermediate; S: Susceptible.

**Figure 2:**
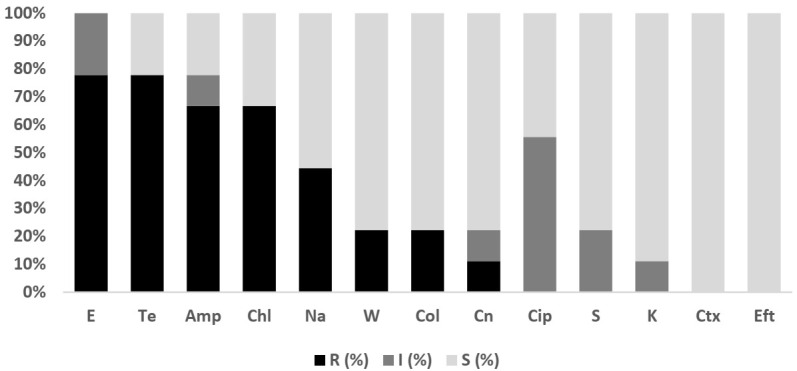
Antibiotic susceptibility pattern of *Salmonella* isolates (*n* = 9) recovered from tilapia production systems on the west coast of Peninsular Malaysia against antimicrobials tested. E, Erythromycin; Amp, Ampicillin; Te, Tetracycline; Chl, Chloramphenicol; Na, Nalidixic Acid; W, Trimethoprim; Col, Colistin; Cn, Gentamycin; Cip, Ciprofloxacin; S, Streptomycin; K, Kanamycin; Ctx, Cefotaxime; Eft, Ceftiofur. R: Resistant; I: Intermediate; S: Susceptible.

**Figure 3:**
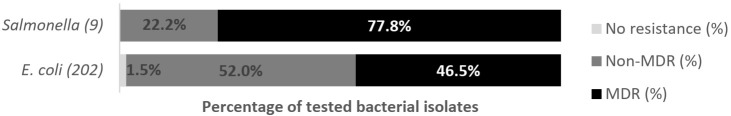
Multi-drug resistance of *E. coli* from tilapia (*n* = 202, fish = 157 and pond water = 45) and *Salmonella* (*n* = 9) recovered from tilapia production systems on the west coast of Peninsular Malaysia. Numbers inside the brackets “()” denote the number of isolates; those on bars indicate percent isolates showing resistance; non-MDR = Resistant against only 1 or 2 classes of antibiotics; MDR = Multidrug resistance.

Overall, 211 isolates comprising *E. coli* (*n* = 202; fish = 157, water = 45) and *Salmonella* spp. (*n* = 9; fish =2, water = 7) were subjected to antibiotic susceptibility testing (AST). The antibiograms of *E. coli* and *Salmonella* isolates are presented in Figure 1 and Figure 2. The level of multi-drug resistance (MDR) for the bacteria is illustrated in Figure 3.

#### 2.3.1. *Escherichia coli*

The antimicrobial resistance profile among *E. coli* isolates from fish and pond water in Figure 1 demonstrated varying levels of resistance against the thirteen antibiotics tested. The highest level of resistance was observed for erythromycin (fish: 98.7% (95% CI = 95.0–99.8); water: 95.6% (95% CI = 83.7–99.2)), ampicillin (fish: 30.6% (95% CI = 23.6–38.5); water: 57.8% (95% CI = 42.3–72)), tetracycline (fish: 31.2% (95% CI = 24.2–39.2); water: 53.3% (95% CI = 38.0–68.0)), and trimethoprim (fish: 29.9% (95% CI = 23.0–37.8); water: 35.6% (95% CI = 22.3–51.3)). Figure 3 shows the level of MDR for isolates from fish (42.7%; 95% CI = 34.8–50.8) (67/157) and pond water (60%; 95% CI = 44.3–74.3) (27/45). Overall, 46.5% (95% CI = 39.5–53.7) (94/202) of *E. coli* isolated from tilapia production systems was resistant to multiple classes of antibiotics; 52.0% (95% CI = 44.9–59) (105/202) was resistant to one or two antibiotic classes and 1.5% (95% CI = 0.3–4.3) (3/202) was susceptible to all the antibiotics tested. 

#### 2.3.2. *Salmonella* spp.

As the number of *Salmonella* isolates was low, isolates from fish and pond water were analyzed together. Figure 2 showed that all *Salmonella* spp. isolates showed resistance or intermediate resistance against the eleven antibiotics tested, while all the isolates were susceptible to ceftiofur and cefotaxime. The highest level of resistance was against erythromycin (77.8%; 95% CI = 40.2, 96.1), tetracycline (77.8%; 95% CI = 40.2, 96.1), ampicillin (66.7%; 95% CI = 30.9, 91.0) and chloramphenicol (66.7%; 95% CI = 30.9, 91.0). A high level of MDR (77.8%; 95% CI = 39–97.2) (7/9) was observed for *Salmonella*, with another 22.2% (95% CI = 2.8–60) (2/9) resistant to one or two antimicrobial agents tested (Figure 3).

### 2.4. Antibiotic Susceptibility according to Species of Bacteria from Farm-Raised Asian Seabass

**Figure 4:**
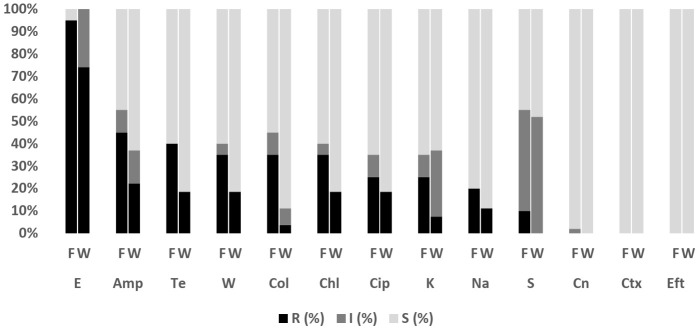
Antibiotic susceptibility pattern of *E. coli* isolates recovered from Asian seabass fish (*n* = 20) and pond water (*n* = 27) on the west coast of Peninsular Malaysia against antibiotics tested. E, Erythromycin; Amp, Ampicillin; Te, Tetracycline; W, Trimethoprim; Col, Colistin; Chl, Chloramphenicol; Cip, Ciprofloxacin; K, Kanamycin; Na, Nalidixic Acid; S, Streptomycin; Cn, Gentamycin; Ctx, Cefotaxime; Eft, Ceftiofur. F: Fish; W: Pond Water; R: Resistant; I: Intermediate; S: Susceptible.

**Figure 5:**
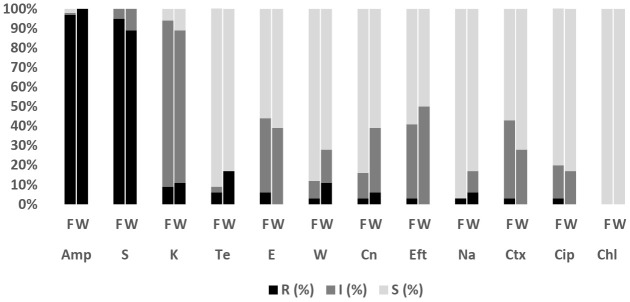
Antibiotic susceptibility pattern of *V. parahaemolyticus* isolates recovered from Asian seabass fish (*n* = 144) and water (*n* = 18) on the west coast of Peninsular Malaysia against antibiotics tested. Amp, Ampicillin; S, Streptomycin; K, Kanamycin; Te, Tetracycline; E, Erythromycin; W, Trimethoprim; Cn, Gentamycin; Eft, Ceftiofur; Na, Nalidixic Acid; Ctx, Cefotaxime; Cip, Ciprofloxacin; Chl, Chloramphenicol. F: Fish; W: Pond Water; R: Resistant; I: Intermediate; S: Susceptible.

**Figure 6:**
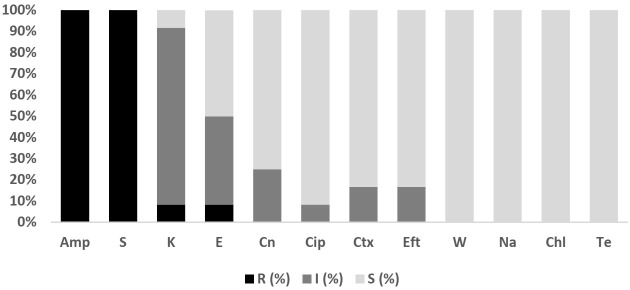
Antibiotic susceptibility pattern of *V. vulnificus* isolates (*n* = 12) recovered from Asian seabass production system on the west coast of Peninsular Malaysia against antibiotics tested. Amp, Ampicillin; S, Streptomycin; K, Kanamycin; E, Erythromycin; Cn, Gentamycin; Cip, Ciprofloxacin; Ctx, Cefotaxime; Eft, Ceftiofur; W, Trimethoprim; Na, Nalidixic Acid; Chl, Chloramphenicol; Te, Tetracycline. R: Resistant; I: Intermediate; S: Susceptible.

**Figure 7:**
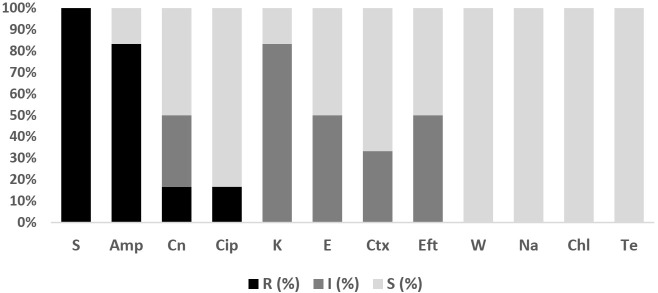
Antibiotic susceptibility pattern of *V. cholerae* isolates (*n* = 6) recovered from Asian seabass production system on the west coast of Peninsular Malaysia against antibiotics tested. Amp, Ampicillin; S, Streptomycin; K, Kanamycin; Te, Tetracycline; Cn, Gentamycin; W, Trimethoprim; E, Erythromycin; Eft, Ceftiofur; Na, Nalidixic Acid; Ctx, Cefotaxime; Cip, Ciprofloxacin; Chl, Chloramphenicol. R: Resistant; I: Intermediate; S: Susceptible.

**Figure 8:**
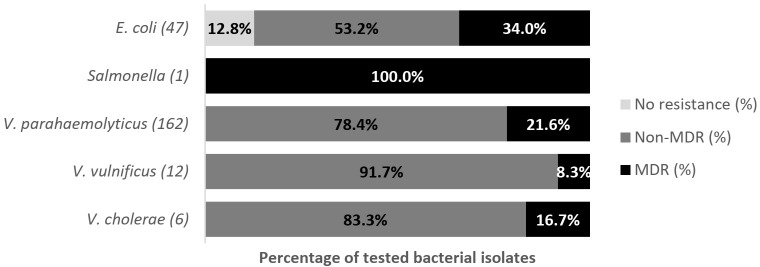
Multi-drug resistance of public-health-significant bacteria recovered from Asian seabass production system on the west coast of Peninsular Malaysia. Numbers inside the brackets “()” denote the number of isolates; those on bars indicate percent isolates showing resistance; non-MDR = only 1 or 2 classes; MDR = Multidrug resistance.

Overall, 228 isolates comprising *E. coli* (*n* = 47; Fish = 20, Water = 27), *Salmonella* spp. (*n* = 1; Fish = 1, Water = 0), *V. parahaemolyticus* (*n* = 162; Fish = 144, Water = 18), *V. vulnificus* (*n* = 12; Fish = 11, Water = 1) and *V. cholerae* (*n* = 6; Fish= 4, Water = 2) were subjected to AST. The antibiograms of *E. coli*, *Salmonella* spp., *V. parahaemolyticus*, *V. vulnificus*, and *V. cholerae* are presented in Figure 4, Figure 5, Figure 6 and Figure 7. The level of MDR for the bacteria isolated is illustrated in Figure 8.

#### 2.4.1. *Escherichia coli*

Figure 4 shows the resistance pattern for *E. coli* in Asian seabass and pond water. The proportion of *E. coli* isolates resistant to erythromycin was 95% (95% CI = 73.1–99.7) for fish and 74.1% (95% CI = 53.4–88.1) for water. Resistance to ampicillin was 45% (95% CI = 23.8–68.0) for fish and 22.2% (95% CI = 9.4–42.7) for water, to tetracycline was 40% (95% CI = 20–63.6) for fish and 18.5% (95% CI = 7.0–38.7) for water, and to trimethoprim was 35% (95% CI = 16.3–59.1) for fish and 18.5% (95% CI = 7.0–38.7) for water. All the isolates were susceptible to cefotaxime and ceftiofur. Figure 8 shows the levels of MDR for isolates from fish (50%; 95% CI = 27.2–72.8) (10/20) and pond water (22.2%; 95% CI = 8.6–42.3) (6/27). Overall, 34.0% (95% CI = 20.9, 49.3) (16/47) of *E. coli* isolated from the Asian seabass production system were MDR, with another 53.2% (95% CI = 38.1, 67.9) (25/47) resistant to one or two antibiotics tested, and 12.8% (95% CI = 4.8, 25.7) (6/47) susceptible to all antimicrobial agents tested (Figure 8).

#### 2.4.2. *Salmonella* spp.

*Salmonella* spp. in Asian seabass fish was 100% resistant toward erythromycin (95% CI = 81, 121.6), streptomycin (95% CI = 81, 121.6), and chloramphenicol (95% CI = 81, 121.6). The *Salmonella* isolated was resistant to three antibiotic classes tested (Figure 8). However, as there was only a single isolate, no meaningful inferences could be made from this finding.

#### 2.4.3. *V. parahaemolyticus*

Figure 5 shows the *V. parahaemolyticus* AMR pattern for isolates from Asian seabass fish and pond water. The highest level of resistance to *V. parahaemolyticus* in fish (96.5%; 95% CI = 92.1–98.8) and pond water (100%; 95% CI = 81.5–100) was for ampicillin, followed by streptomycin in fish (95.1%; 95% CI = 90.2–98) and pond water (88.9%; 95% CI = 65.3–98.6). In addition, the isolates were susceptible to chloramphenicol. Figure 8 shows the levels of MDR for isolates from fish (20.8%; 95% CI = 14.5–28.4) (30/144) and pond water (27.8%; 95% CI = 9.7–53.5) (5/18). Overall, 21.6% (95% CI = 15.5, 28.7) (35/162) of *V. parahaemolyticus* isolated from the Asian seabass production system were MDR while 78.4% (95% CI = 71.2, 84.5) (127/162) were resistant to one or two antibiotics (Figure 8). All isolates were non-susceptible to at least one of the twelve antimicrobial agents tested.

#### 2.4.4. *V. vulnificus*

None of the tested *V. vulnificus* isolates were susceptible to the action of ampicillin (100%; 95% CI = 81.4, 121.6) or streptomycin (100%; 95% CI = 81.4, 121.6). The isolates were sensitive to trimethoprim, nalidixic acid, chloramphenicol and tetracycline (Figure 6). The MDR of *V. vulnificus* was relatively low at 8.3% (95% CI = 0.2, 38.5) (1/12), while 91.7% (95% CI = 61.5, 99.8) (11/12) were resistant to one or two antibiotics tested (Figure 8).

#### 2.4.5. *V. cholerae*

Complete resistance was observed towards streptomycin (100%; 95% CI = 81.4, 121.6), while the resistance rate was high to ampicillin (83.3%; 95% CI = 54, 112) (Figure 7). Figure 8 shows that 16.7% (95% CI = 0.4, 64.1) of *V. cholerae* isolated were MDR while 83.3% (95% CI = 35.9, 99.6) were resistant to one or two antibiotics tested.

### 2.5. Differences between Resistance Profile of E. coli Isolates from Tilapia and Asian Seabass

Table 3 illustrates the antimicrobial resistance profiles among *E. coli* isolated from tilapia and the Asian seabass production system. The antimicrobial resistance profile for Table 3 was combined both from fish and pond water and the data presented in the Supplementary Materials Table S1. *E. coli* isolates were most frequently resistant to erythromycin (83 to 98%), ampicillin (32 to 37%) and tetracycline (28 to 36%) in both tilapia and Asian seabass (Table 3). In general, *E. coli* isolates from tilapia showed higher-level resistance toward eight antimicrobial agents tested, with 98% resistance toward erythromycin. In contrast, *E. coli* isolated from Asian seabass showed slightly higher resistance to chloramphenicol, ciprofloxacin, nalidixic acid and kanamycin (14.9 to 25.5%) than *E. coli* from tilapia for the same antibiotics. Statistically significant differences between the AMR level in *E. coli* isolates from tilapia and Asian seabass were recorded for erythromycin and streptomycin, while there were no significant differences in proportion of MDR among *E. coli* isolated from tilapia and Asian seabass (χ^2^ = 2.413 *p* = 0.120).

### 2.6. Differences between Resistance Profile of E. coli Isolates from Aquaculture and Livestock

Table 4 shows that *E. coli* isolated from the surveillance of live broilers, layers and pigs [21] in intensive farms recorded markedly higher levels of resistance against several antibiotics. The highest resistance in livestock was against ampicillin and tetracycline, and the lowest was against gentamycin, cefotaxime and ceftiofur where the ranges were more comparable with the low readings for fish in the present study.

## 3. Discussion

AMR surveillance involving aquaculture production has been included as an important agenda in the AMR action plan in many countries, including Malaysia. There are very few reports on AMR among *E. coli* and *Salmonella* from cultured tilapia and Asian seabass, the two most highly consumed aquaculture products in Malaysia [3,4]. Hence, the discussion will incorporate local and regional information where available.

### 3.1. E. coli

#### 3.1.1. The Resistance Pattern of *E. coli* in Tilapia and Asian Seabass

The resistance patterns for all tested antibiotics for *E. coli* from tilapia and Asian seabass and their environment are comparable. Resistance was highest for erythromycin, tetracycline, ampicillin and trimethoprim and lowest for cefotaxime, ceftiofur and gentamycin. Significant differences were observed for resistance against erythromycin and streptomycin (Table 3). Of note is the resistance level of *E. coli* to colistin at 17–18.3%, which was lower than for *E. coli* recovered from fish from the market in India (30.9%) [22] and *E coli* isolated from farmed fish (92.9%) in China [23]. However, we found colistin resistance in this study to be higher than that detected in *E. coli* from a previous report on various fish, clam, cockle and bivalve farms in Malaysia (7.3%) [2] and from pangasius catfish (<10%) from Vietnam [24].

The very high level of resistance to erythromycin supports the previous report by [2]. However, slightly lower levels of resistance of *E. coli* to tetracycline (18.2%), chloramphenicol (10%) and ampicillin (15%) were reported in that study. The highest resistance observed for erythromycin among antibiotics tested was consistent with that recorded in *E. coli* isolated from farm-raised tilapia in Bangladesh (81.25%) and Africa (72.7%) [25,26]. Sensitivity to ciprofloxacin and gentamycin was reported in an African study [25], as in this study. In contrast, studies from India (ciprofloxacin; 60.5%) [22] and Vietnam (ciprofloxacin; 78.6% and gentamycin; 88.3%) [27] documented higher levels of resistance to the aforementioned antibiotics. Studies from Vietnam and India also consistently reported higher resistance against several other antibiotics tested in this study. In Vietnam, *E. coli* isolated from catfish and tilapia recorded high resistance against tetracycline (88.1%), chloramphenicol (78.6%), ciprofloxacin (78.6%), nalidixic acid (92.9%), gentamycin (88.3%), streptomycin (88.1%) and kanamycin (76.2%) [27]. In India, higher levels of resistance to streptomycin (95%), trimethoprim (76.5%), ciprofloxacin (60.5%), chloramphenicol (21%), and colistin (30.9%) were recorded from cultured fish [26]. The same study [22] observed higher MDR (92.6%) among *E. coli* isolates compared to our study. Unfortunately, there was no information about the MDR level in *E. coli* isolates from the afore-cited studies from Vietnam, Bangladesh and Africa to compare with our findings. 

Generally, *E. coli* from the Asian seabass production system in this study showed slightly lower resistance levels to the majority of antibiotics tested compared to tilapia. *E. coli* resistance to kanamycin in this study was comparable to that of Asian seabass fingerlings from Malaysia [28]. Low resistance levels toward streptomycin (31%), kanamycin (19%), and nalidixic acid (22%) were reported in *E. coli* isolated from a fish farm along a mangrove forest reserve in Perak, Malaysia [29]. In other parts of the world, a study of Mullet fish from marine farms in Egypt recorded higher prevalence levels of resistance toward streptomycin (100%), but the isolates were sensitive to nalidixic acid [30]. Very low occurrence of resistance to cefotaxime among *E. coli* isolates in the present study was in contrast to the high-level resistance to third-generation cephalosporin (cefotaxime) from cultured fish in Egypt (86.5%) [30] and from the aquaculture environment in Singapore (ceftazidime, 97.5%) [31].

#### 3.1.2. The Comparison of Resistance with Livestock

Terrestrial anthropogenic sources are known to influence the presence of *E. coli* and antibiotic availability in aquatic systems; we compared our findings to those from local AMR surveillance in livestock raised in intensive farms. Although the comparison was not conclusive given that the site of sampling was not matched, it gave some indication of the one health interconnections of AMR across systems. Unfortunately, erythromycin was not included for livestock surveillance work for comparison. *E. coli* isolates recovered from aquacultures in this study had lower levels of resistance to the various antimicrobials compared to those reported from live poultry and pigs [21]. In comparison, *E. coli* isolated from diseased ruminants [13] recorded higher levels of resistance against tetracycline (52.2%; 95% CI = 39.9–64.2), gentamycin (68.2%; 95% CI = 45.1–85.3), and streptomycin (82.5%; 95% CI = 69.7–90.9) as compared to data from this study. There were similarities in the pattern of resistance between *E. coli* from livestock and farmed fish in this study, albeit lower resistance levels were observed across antibiotics amongst isolates from aquaculture. There are a number of explanations for these observations. It is possible that dilution and natural degradation of the antibiotics in the aquatic system [32,33] cause decreasing antibiotic concentration, leading to a weakened spread of resistance. Large spatial distances have been found to incapacitate transfer of resistance elements [33]. Antibiotic degradation is an important process affecting the fate of antibiotic-resistant bacteria and antibiotic resistance genes (ARGs) in the freshwater environment [34]. Other environmental physicochemical parameters may play additional role in influencing the prevalence of resistance [35].

The emergence of MDR amongst foodborne pathogens is a great public health challenge [36]. In this study, we found that the frequency of MDR in *E. coli* in an Asian seabass and tilapia production system level to be between 34% (95% CI = 20.9–49.3) and 46.5% (95% CI = 39.5–53.7). This level was lower compared to MDR of *E. coli* isolates recovered from diseased ruminants and non-ruminants (67.4% and 72.2%, respectively) [13], broiler (100%) [37] and poultry (80.2%) [38]. The high MDR level of *E. coli* isolates in livestock is consistently reported in multiple studies from Southeast Asia countries: in Thailand, 84.3% and 48% of isolates from dairy farm and pigs, respectively [39,40], in Vietnam, 53% and 81.3% of isolates from dairy calves and chicken (layers and broilers), respectively [41,42], and in Indonesia, 57.3% and 100% of isolates from pigs and broilers, respectively [43,44]. The lower MDR level among *E. coli* isolates in aquaculture is possibly due to the infrequent use of antibiotics in aquaculture production but may also be the result of a decreased antibiotic concentration and transformation of antibiotics in the water environment [32]. The concentration of antimicrobials in the surface water is vulnerable to external environmental influences, including dilution of antibiotics by leaching or water current, adsorption of particles and photo degradation [35]. In addition, the elimination of antibiotics and ARGs in the water environment can result from biotic (biodegradation by bacteria and fungi) and non-biotic (hydrolysis, photolysis, oxidation and reduction) processes influenced by environmental, chemical and physical variables [32,34,45]. An example is oxytetracycline degradation in sediment in water after 75 days under anaerobic and 47 days under aerobic conditions [46].

### 3.2. Salmonella *spp.*

Similar to *E. coli*, *Salmonella* sp. demonstrated high resistance levels to most antibiotics tested, such as erythromycin, tetracycline, and ampicillin, as well as non-susceptibility against streptomycin, ciprofloxacin, and kanamycin. Contrary to this finding, a previous study on *Salmonella* in catfish, tilapia and pond water from Malaysia identified lower levels of resistance against chloramphenicol (37.2%) and tetracycline (67.4%) [9]. Nevertheless, a study conducted in the Malaysian state of Sabah found that *Salmonella* isolated from cultured catfish had high (100%) resistance to tetracycline, although it was susceptible to trimethoprim [47]. In other parts of the world such as in Nigeria, a slightly higher level of resistance of *Salmonella* from cultured fish against streptomycin (43.5%) and trimethoprim (21.7%) [48] was reported. Similarly, higher resistance to streptomycin (98.6%), trimethoprim (79.2%), chloramphenicol (25%), and colistin (25%) was recorded from cultured fish in India [22]. The resistance pattern of ampicillin, tetracycline and chloramphenicol (≥70%) of *Salmonella* spp. isolated from farm-raised tilapia and catfish as well as pond water in Africa [49] was similar to our observation in this study. The level of MDR (77.8%) in the present study was lower than that reported from cultured fish (88.9%) in India [22] but higher than recorded from cultured tilapia and catfish in Africa (12.2%) [49].

The present study also observed resistance of non-typhoidal *Salmonella*, *S. typhimurium* (*n* = 2) to fluoroquinolone and nalidixic acid (50%; 95% CI = 1.3–98.7) and non-susceptibility against ciprofloxacin (100%; 95% CI = 15.8–100). This is of particular public health concern since it is the drug of choice to treat invasive salmonellosis in adults [50]. Moreover, nalidixic acid and ciprofloxacin are listed under veterinary critically important antibiotics (VCIA) on the OIE antibiotic list that are of particular importance in treating diseases in animal production [51]. On a positive note, all *Salmonella* isolates in this study were susceptible to some of the highest priority critically important antimicrobials under the WHO as well as VCIA under OIE, including third-generation cephalosporins such as cefotaxime and ceftiofur. 

### 3.3. Vibrio *sp.*

Unlike *E. coli* and *Salmonella* in this study that demonstrated the highest resistance to erythromycin, the highest resistance among *V. parahaemolyticus* isolates was to ampicillin (96.9%) and streptomycin (94.4%). This resistance level was also observed in a previous study on farm-raised marine fish (77–84%) [19] and Malaysian seafood (84.7%) [20]. In the present study, resistance to ampicillin and streptomycin was higher (94.4–96.9%) as compared to that encountered in Poland, 75% and 68.3%, respectively [52], and in China, 79.6% and 68.3%, respectively [53]. Similarly high resistance to ampicillin (100%) was reported among the isolates from fish cultured in Egypt [54].

Limited reports on MDR on *V. parahaemolyticus* in seafood from the local fresh market have suggested very high MDR levels (90.83%) [20] as compared to results from the present study. The MDR level was also lower than those identified in oyster and shrimp (68.38%) from China [53], but much higher than reported in marine fish and shellfish (1.5%) obtained from the Polish market [52]. 

Among the *Vibrio* species in this study, the highest level of resistance to ampicillin was observed in *V. vulnificus* (100%) and *V. cholerae* (83.3%). These findings are consistent with previous work where the ampicillin-resistant *V. vulnificus* was reported at 100% from aquaculture products and aquaculture systems in India and Nigeria [55,56]. Two separate studies conducted in Malaysia and Qatar documented that *V. vulnificus* was highly resistant to ampicillin in cultured fish (64.5%) and displayed the second-highest level of resistance in cockles as well as clams (70%) [57]. In previous research, *V. vulnificus* resistance to streptomycin had been reported in cultured marine fish from Malaysia (15%) [19], as had *V. cholera* resistance in Malaysian farmed fish (25%) [58]. High prevalence of MDR in *V. vulnificus* (95%) was reported in cockles and clams isolated both from Malaysia and Qatar [57], while a lower prevalence of MDR for *V. cholerae* (1.8%) was recorded in inland saline aquaculture in India [59].

All *V. vulnificus* and *V. cholerae* isolates were susceptible to tetracycline, ciprofloxacin as well as third-generation cephalosporins. These are the recommended antibiotics by the United States Centre for Diseases Control and Prevention (CDC) for human clinical treatment of *Vibrio* species infection [10,60,61]. *V. parahaemolyticus*, *V. vulnificus* and *V. cholerae* are the most important human pathogens that originate from aquatic and marine habitats [62]. Low rates of tetracycline, ciprofloxacin and cefotaxime resistance against *V. parahaemolyticus* observed in the present study is comparable with results from studies from Malaysia (tetracycline 16%), Korea (ciprofloxacin 6.8%), and Saudi Arabia (cefotaxime 13.3%) that documented low resistance of the aforementioned antibiotics in aquaculture [19,63,64].

### 3.4. The Resistance Pattern for E. coli in Tilapia and Asian Seabass

*E. coli* is considered a sentinel for AMR in a wide range of animal species, hence a suitable candidate in comparing resistance between two different groups [13,65]. In addition, *E. coli* is regarded as a target microorganism to be investigated for AMR surveillance under the Malaysia Action Plan on AMR in the aquaculture sector [2]. Across aquaculture products and species of bacteria in this study, resistance to erythromycin was consistently highest as compared to other tested antibiotics. This observation has also been reported in other aquaculture studies, such as from farmed tilapia and Mrigal carp (*Cirrhinus mrigala*) in Bangladesh (81.2%), farmed tilapia in Africa (72.7%) and various farms in Malaysia (90.7%) [2,25,26]. Statistically significant differences were observed for the AMR of *E. coli* isolates from tilapia and Asian seabass against erythromycin and streptomycin. One possible reason is the wider application of antibiotics in tilapia, surpassing that for brackish water and marine organisms such as shrimp, trout, and salmon [66]. Unfortunately, no data on antibiotic use in aquaculture in Malaysia are available. Based on a report by [67], tilapia production in Malaysia had suffered more disease outbreaks, resulting in relatively higher mortality and economic losses, compared to Asian seabass production. In Malaysia, bacterial diseases are a major burden in tilapia production; infection with *Streptococcus* sp. in general, and co-infection of *S. agalactiae* with tilapia lake virus (TiLV) are responsible for high mortality rate [68,69,70,71], leading to a decrease in tilapia production between 2012–2018 [72]. According to [73], erythromycin is commonly used for the treatment of streptococcal diseases in fish and is considered a drug of choice that can effectively curb streptococcal infection [74]. Erythromycin and oxytetracycline are frequently incorporated into the fish pellet for streptococcosis treatment in tilapia as well as being used as a prophylactic agent in healthy fish [73]. 

Unfortunately, as with other livestock, antimicrobials usage in the aquaculture industry is not monitored [2] and, therefore, accurate data are not available. Erythromycin is one of the antibiotics allowed by OIE for use in aquaculture [75] and is a veterinary drug registered with the National Pharmaceutical Control Bureau (NPCB) of the Ministry of Health, Malaysia [76]. The consistently high resistance level of erythromycin across bacterial species in tilapia and Asian seabass may likely be due to a large volume of erythromycin reaching water bodies of the aquatic system from the extensive usage not just in aquaculture but also in its use in human health and in animal production [77,78]. It is also possible that the resistant nature of erythromycin to biodegradation during biological treatment [79], persistence in the wastewater treatment process [80] and incomplete removal under the activated sludge process of the saline and freshwater sewage system as compared to other antibiotics [81]. This may facilitate prolonged selective pressure to bacteria in the aquatic environment. In addition, macrolides are reported to be less susceptible to hydrolysis, one of the most important pathways for abiotic degradation of antibiotics, thereby allowing them to persist longer in the environment [82]. Antibiotic persistence in the aquatic system is defined based on its half-life value [83]. Erythromycin has a significantly longer half-life in surface water (<17 days) compared to other antibiotics tested, such as ciprofloxacin, trimethoprim, tetracycline [83,84] and chloramphenicol in pond water [85]. In groundwater or soil/sediment, the half-life can be much longer due to scarcity of sunlight and aerobic conditions [83]. Schlüsener & Bester [86] reported the half-life of erythromycin in the soil at about 20 days. Erythromycin is also easily absorbed in soil components and the process of absorption enables erythromycin to persist in the aquatic sediment [87,88], thus increasing the possibility for further adaptation over time, human exposure risk, and environmental transmission [89].

### 3.5. Resistance to Colistin

Resistance to colistin is a major public health concern since the antibiotic is considered as the last resort drug against multidrug-resistant Gram-negative bacteria causing life-threatening infections in humans [90,91]. Malaysia has recently banned the use of colistin in animals [92]. This study found that the colistin resistance levels of *E. coli* and *Salmonella* spp. were about 18.1% and 20%, respectively. Although there is a paucity of information from Malaysia, there is evidence that colistin-resistant *E. coli* and *mcr*-gene-containing bacteria circulate in the aquaculture and aquatic systems. For instance, colistin-resistant *E. coli* (7.3%) has been recorded in various aquaculture farms in Peninsular Malaysia [2] and *E. coli* harboring *mcr*-1 was observed in the pond water [93] and water system in Malaysia [94]. However, none of the Malaysian studies investigated *mcr*-1 in *Salmonella*. Many countries such as Lebanon, Vietnam, Spain and China have reported the *mcr*-1 gene in *E. coli* and *Salmonella enterica* in aquaculture [95,96,97,98]. Moreover, a study from China observed that the *mcr*-1 *E. coli* isolates from integrated aquaculture farms were genetically related to those from human sources in the farm regions [23]. Hence, not only is aquaculture susceptible to terrestrial-related activities, it has been suggested that aquaculture can promote, select, and mobilize *mcr* genes to terrestrial bacteria by horizontal gene transfer to yield colistin-resistant human pathogens [23,99,100].

Our study should be interpreted with caution because of several limitations. A major limitation is sampling bias because the fish farms were not selected randomly but were chosen based on the willingness of farmers to participate in this study. As sampling was conducted only once for each farm, we could not capture variations that might arise from changes in water parameters over time and season. In addition, the aqua farms in this study were only from the central region of the west coast of Peninsular Malaysia. Therefore, the findings from this study may not represent the aquaculture farms in Peninsular Malaysia

## 4. Materials and Methods

### 4.1. Study Areas

Malaysia (comprising Peninsular Malaysia and East Malaysia) has a total coastline of 4675 km [4]. Given the extended coastline, brackish water aquaculture dominates the fish farming industry in Malaysia, covering an area of 17,357 ha [101]. Freshwater aquaculture is gaining popularity and covers an area of 7936 ha spread throughout the country [4]. The preferred systems to produce fish both in brackish water and freshwater environments are pond and cage systems. Pond systems occupy an area of 7525.43 ha and 5642.31 ha for brackish water and freshwater aquaculture, respectively [4]. The present study is conducted on the west coast of Peninsular Malaysia, specifically in four states, viz. Selangor, Negeri Sembilan, Melaka, and Perak. The map of the study area is presented in the Supplementary Materials Figure S1.

### 4.2. Sample Size

The study population comprised cultured red hybrid tilapia (*Oreochromis* spp.) and Asian seabass (*Lates calcarifer*, Bloch 1970) located in aquaculture farms in the central region of Peninsular Malaysia. The sample size was calculated using the formula for simple random sampling for a large population that was previously described by [102] and using 95% confidence interval (CI) and 10% absolute precision. Assuming an expected prevalence for *Salmonella* of 30% as previously reported in catfish in Malaysia [9] and *Vibrio* of 50% as the previous data in grouper fish in Malaysia [103], the number of tilapia and Asian seabass fishes for each state was 81 and 96, respectively. The list of farms was obtained from the Department of Fisheries (DOF) of each sampled state, and available farms were selected based on the willingness of farmers to participate in the study. In total, the study included 19 grow-out tilapia farms in Selangor (*n* = 6), Negeri Sembilan (*n* = 11) and Melaka (*n* = 2) and 13 grow-out Asian seabass farms in Selangor (*n* = 4), Negeri Sembilan (*n* = 1) and Perak (*n* = 8).

### 4.3. Study Design

A cross-sectional study was conducted whereby all samples, as well as information about production systems, were collected during sampling. Thirty-two farms were selected from the list of aquaculture farms recommended by state-level Department of Fisheries (DOF). Farmers were called to explain about the study and were invited to participate with assurance of confidentiality. Participation in the study was voluntary.

The earthen pond production system was the predominant system that was mostly adopted for tilapia farming. A few tilapia farms used higher technology such as paddle wheel aerator for oxygen supply in the pond. In Malaysia, the pond system is the preferred system used to raise freshwater commodities [4]. The cage culture system is not commonly adopted to raise tilapia in the study area. On the other hand, Asian seabass were predominantly raised in floating cages with the water sourced from estuaries, rivers and the open sea.

### 4.4. Sample Collection from Farms

Sample collection was performed from February 2019 to December 2019. All fishes collected were at the market age. For tilapia, the market age ranges from 4–6 months, while for Asian seabass, the range is 8–10 months. The fishes were caught using cast-nets. Five hundred-milliliter (500 mL) water samples were collected from aquaculture water using sterile glass bottles. Water samples were collected at a depth 0–30 cm below the water surface, close to outlet pipe for earthen ponds and at the edge of cages for the cage system. The fishes and water samples were immediately packed into cool boxes and transported to the Veterinary Public Health Laboratory, Faculty of Veterinary Medicine, Universiti Putra Malaysia, and immediately processed for bacterial culture and identification. This study was approved by the Institutional Animal Care and Use Committee of University Putra Malaysia (UPM/IACUC/AUP-R009/2019).

### 4.5. Isolation and Identification of E. coli

The isolation of *E. coli* from tilapia and Asian seabass adopted the method of [104,105]. Two grams of fish intestine were incorporated into 18 mL of Buffered Peptone Water (BPW) and incubated at 37 °C for 18–24 h. Briefly, samples were streaked and cultured with Levine Eosin Methylene Blue (L-EMB agar, Oxoid) agar and MacConkey Agar (MCA, Oxoid), then incubated at 37 °C for 18–24 h. Presumptive colonies were identified using a series of biochemical tests. *E. coli* from water samples were isolated by using the membrane filtration technique (MFT) [33]. A hundred milliliters of water samples was filtered through a 0.45 µm nitrocellulose filter (47 mm diameter). Then, the filter was transferred on to Chromocult Coliform agar (Merck) and incubated at 37 °C for 18–24 h. Three suspected *E. coli* colonies were randomly picked and subjected to further biochemical examinations [104]. *E. coli* (ATCC 25922) was used as the reference strain.

### 4.6. Isolation and Identification of Salmonella

The isolation of *Salmonella* was carried out according to the protocols of the World Organization for Animal Health (OIE), Manual of Diagnostic Test and Vaccines for Terrestrial Animal (www.oie.net, accessed on 23 July 2021). Briefly, 2 g of intestine were pre-enriched in Buffer Peptone Water (BPW) and followed by enrichment in Rappaport Vassiliadis (RV) Enrichment broth (Oxoid, UK). Following incubation, a loopful of the culture was streak-plated onto Xylose Lysine Deoxycholate (XLD, Oxoid, UK) and Briliant Green Agar (BGA, Oxoid, UK). The colony with a typical morphology, according to the assay manufacturer’s instructions, was considered as presumptive *Salmonella.* The isolation of *Salmonella* from water samples adopted the method from [106]. A hundred milliliters of water sample was filtered through 0.45 µm nitrocellulose filters (47 mm diameter) and was pre-enriched in BPW incubated at 30 °C for 4 h. After incubation at 42 °C for 24 h, a loopful of the broth was streaked onto XLD and BGA. The suspected colonies were confirmed by using the biochemical test and polyvalent O and H antisera according to the manufacturer’s instructions [107]. Finally, the isolate was screened for *Salmonella* genus, *S. typhimurium* and *S. enteritidis* using the PCR method described by [108]. The primers used and PCR conditions are described in the Supplementary Materials Table S2. 

### 4.7. Isolation and Identification of Vibrio

Two grams of intestine were mixed with 18 mL enrichment broth, alkaline peptone water (APW, Oxoid, UK) for 16–18 h at 30 °C [109,110]. Then, surface growth was collected with an inoculating loop and streaked onto thiosulfate-citrate-bile salt-sucrose agar (TCBS, Oxoid, UK). The plates were incubated for 24 h at 30 °C. The presumptive colonies for *Vibrio* sp. were selected based on the manufacturer’s instructions. *Vibrio* sp. was isolated from a water sample by using the filter method [106,110]. A hundred milliliters of water sample was filtered through 0.45 µm nitrocellulose filters (47 mm diameter). Then, the filters were pre-enriched in 9 mL APW (Oxoid, UK) and the surface aliquots were streaked for isolation onto TCBS agar and incubated at 30 °C for 24 h. Finally, the presumptive colonies were subjected to bacterial identification using biochemical tests and examined further by the multiplex PCR method [111] for *V. parahaemolyticus*, *V. vulnificus* and *V. cholerae.* The primers and PCR conditions are presented in the Supplementary Materials Table S3. The confirmed isolates were stored in glycerol (Tryptone Soya Broth with 50% glycerol at −40 °C) for further analysis.

### 4.8. Antimicrobial Susceptibility Test

The susceptibility to antibiotics of *E. coli*, *Salmonella* and *Vibrio* was tested for 13 antibiotics. Twelve antibiotics were tested using disc diffusion (concentration in µg): cefotaxime (30 µg), ceftiofur (30 µg), ciprofloxacin (5 µg), gentamycin (10 µg), chloramphenicol (30 µg), streptomycin (10 µg), ampicillin (10 µg), trimethoprim (5 µg), erythromycin (15 µg), nalidixic acid (30 µg), kanamycin (30 µg), and tetracycline (30 µg). Colistin was tested using the broth microdilution method (BMD). Antibiotics were selected based upon the recommendation by WHO and OIE for antimicrobial use in both human and food-producing animals [75,112]; the selection was consistent with Malaysia’s Antimicrobial Resistance Integrated Surveillance recommendations.

The disk diffusion method for *E. coli* and *Salmonella* was performed according to the Clinical and Laboratory Standards Institute (CLSI) guidelines [113], whereas that for *Vibrio* was conducted according to the CLSI guideline [114,115]. BMD for colistin was performed according to the CLSI guideline [113,116]. BMD is the only approved method for minimum inhibitory concentration (MIC) determination as specified by the European Committee on Antibiotic Susceptibility testing (EUCAST) and the CLSI [113,116,117]. 

### 4.9. Data Analysis

Descriptive statistics were performed to determine the prevalence of *E. coli*, *Salmonella* and *Vibrio* among samples. Separate analyses were performed between isolates from fish and pond water whenever the total number of isolates was more than 10 for each. If the number of isolates was small, the isolates from the fish and their pond water were combined. 

Antimicrobial sensitivity test data of *E. coli* and *Salmonella* isolates from tilapia and Asian seabass were analyzed separately in WHONET 5.6 [118,119]. The CLSI interpretative criteria for disk diffusion susceptibility testing for *Vibrio* was carried out as per CLSI standards [115,120,121]. A chi-square test was used to compare differences of AMR pattern between tilapia and Asian seabass. The frequency of MDR to bacteria between tilapia and Asian seabass was tabulated and compared. The AMR pattern from this study was also compared to the AMR surveillance data from livestock [21]. All the statistical analyses were performed using the SPSS (version 26.0, IBM, Armonk, NY, USA: IBM Corp.) at significance level α = 0.05.

## 5. Conclusions

This study provides an overall picture of the resistance trends of clinically important bacteria *E. coli*, *Salmonella*, *V. parahaemolyticus*, *V. vulnificus*, and *V. cholerae* isolated from aquaculture production on the west coast of Peninsular Malaysia. Significantly higher proportions of resistance to erythromycin and streptomycin among *E. coli* isolates were observed in tilapia compared to Asian seabass. Nevertheless, the MDR level did not significantly differ between the two groups of fishes. The findings highlighted the high resistance level of bacteria isolated towards antibiotics categorized as a priority and critically important for human use and as veterinary critically important drugs for food-producing animals, indicating important risk to public and animal health. Aquaculture is an emerging industry that will continue to grow. Hence, appropriate intervention of antibiotic use is required to ensure the continuous efficacy of antibiotics for animal and human health and the sustainability of the industry. 

## Figures and Tables

**Table 1:** *E. coli* and *Salmonella* spp. recovered from the aquaculture systems in the Malaysian states of Selangor, Negeri Sembilan, Melaka and Perak.

Sample Types	Total	Positive (95% CI)	
		*E. coli*	*Salmonella* sp.
Tilapia	312	139 (44.5; 39.4–50.4)	2 (0.6; 0.2–1.5)
Asian seabass	265	14 (5.3; 2.6–7.9)	1 (0.4; 0.3–1.09)
Tilapia pond water ^1^	19	19 (100; 82.4–100)	3 (15.7; 3.4–39.6)
Asian seabass pond water ^1^	13	8 (61.5; 31.6–86.1)	0 (0)
Overall	609	181 (29.7; 26.1–33.5)	6 (0.9; 0.4–2.1)

^1^ One 500 mL sample of water was collected from each farm.

**Table 2:** *Vibrio* sp. recovered from the aquaculture systems in the Malaysian states of Selangor, Negeri Sembilan, Melaka and Perak.

Sample Type	Total	Positive (95% CI)		
		*V. parahaemolyticus*	*V. vulnificus*	*V. cholera*
Tilapia	312	NA ^2^	NA ^2^	0 (0)
Asian Seabass	265	94 (35.5; 29.7–41.3)	6 (2.3; 0.8–4.8)	5 (1.9; 0.61–4.3)
Tilapia water ^1^	19	NA ^2^	NA ^2^	0(0)
Asian seabass water ^1^	13	6 (46.2; 19.2–74.8)	1 (7.7; 0.2–36)	1 (7.7; 0.2–3.6)

^1^ One 500 mL sample of water was collected from each farm. ^2^ NA: Not applicable because *V. parahaemolyticus* and *V. vulnificus* are strictly halophiles.

**Table 3:** Antimicrobial resistance profile between *E. coli* isolates from Tilapia and Asian seabass in the west coast of Peninsular Malaysia.

Antimicrobial Agent	Resistance% (95% CI)	
	Tilapia	Asian Seabass
Ampicillin	36.6 (30.0–43.7)	31.9 (19.5–47.2)
Chloramphenicol	15.3 (10.8–21.2)	25.5 (14.4–40.6)
Ciprofloxacin	16.8 (12.1–22.8)	21.3 (11.2–36.1)
Colistin	18.3 (13.4–24.5)	17 (8.1–31.3)
Cefotaxime	0.5 (0–3.2)	0 (0.0–9.4)
Erythromycin	98 (94.6–99.4)	83 (68.7–91.9) *
Gentamycin	4.5 (2.2–8.6)	0 (0.0–9.4)
Kanamycin	13.9 (9.6–19.6)	14.9 (6.7–28.9)
Nalidixic Acid	9.9 (6.3–15.1)	14.9 (6.7–28.9)
Streptomycin	19.8 (14.7–26.1)	4.3 (0.8–15.8) *
Tetracycline	36.1 (29.6–43.2)	27.7 (16.1–42.9)
Ceftiofur	0 (0.0–2.3)	0 (0.0–9.4)
Trimethoprim	31.2 (25.0–38.1)	25.5 (14.4–40.6)

* Significant difference between tilapia and Asian seabass production systems, *p* < 0.05.

**Table 4:** Antimicrobial resistance profile of *E. coli* isolates from Tilapia and Asian seabass on the west coast of Peninsular Malaysia compared with livestock.

Antimicrobial Agent	Resistance% (95% CI)			
	Fish ^1^	Layer ^2^	Broiler ^2^	Pig ^2^
Erythromycin	95.6 (92–97.7)	NA	NA	NA
Ampicillin	35.7 (29.8–42)	61 (46.6–78.4)	92 (74.2–102.8)	84 (67–104)
Tetracycline	34.5 (28.7–40.8)	78 (61.6–97.3)	94 (75.9–115)	84 (67–104)
Trimethoprim	30.1 (24.6–36.3)	NA	NA	NA
Colistin	18.1 (13.6–23.6)	NA	NA	NA
Chloramphenicol	17.3 (12.9–22.7)	32 (21.9–45.2)	80 (63.4–99.5)	76 (59.9–95.1)
Ciprofloxacin	17.7 (13.3–23.1)	22 (13.8–33.3)	48 (35.4–63.4)	16 (9.1–25.9)
Streptomycin	16.9(12.6–22.3)	24 (15.4–35.7)	56 (42.3–72.2)	60 (45.8–77.2)
Kanamycin	14.1 (10.1–19.2)	NA	NA	NA
Nalidixic Acid	10.4 (7–15)	NA	NA	NA
Gentamycin	3.6 (1.8–7.0)	4 (1.1–10.4)	31 (21.1–44)	16 (9.1–25.9)
Cefotaxime	0.4 (0.0–2.6)	9 (4.1–17.1)	15 (8.4–24.7)	7 (2.8–14.4)
Ceftiofur	0 (0.0–1.9)	4 (1.1–10.4)	8 (3.5–15.8)	7 (2.8–14.4)

^1^*E. coli* isolated from tilapia and Asian seabass (*n* = 249). ^2^ *E. coli* isolated from layers, broilers and pigs: Surveillance of AMR by the [21]. NA: Erythromycin, colistin, kanamycin, nalidixic acid and trimethoprim were not tested in the surveillance of AMR by the [21].

## Data Availability

The data presented in this study are available in the manuscript.

## Supplementary Materials

The following are available online at https://www.mdpi.com/article/10.3390/antibiotics11020136/s1, Figure S1. Map of study area indicating its four states in the west coast of peninsular Malaysia; Perak, Selangor, Negeri Sembilan and Melaka. Table S1: Antimicrobial Resistance Profile between *E. coli* isolates from Fish and Water Pond in Tilapia and Asian seabass. Table S2. Primers and PCR conditions for *Salmonella*. Table S3. Primers and PCR conditions for *Vibrio*.
