# Supplementary material for: Prevalence and Antimicrobial Resistance of Escherichia coli, Salmonella and Vibrio Derived from Farm-Raised Red Hybrid Tilapia (Oreochromis spp.) and Asian Sea Bass (Lates calcarifer, Bloch 1970) on the West Coast of Peninsular Malaysia
Source: Antibiotics (Basel). 2022 Jan 20;11(2):136. doi: 10.3390/antibiotics11020136 (PMC8868497; doi:10.3390/antibiotics11020136)
Supplement: Supplementary file 1 [file antibiotics-11-00136-s001.zip › antibiotics-1508062-supplementary.pdf]

Supplementary Materials

Table S1. Antimicrobial Resistance Profile between *E. coli* isolates from Fish and Water Pond in Tilapia and Asian seabass

**Antimicrobials**	**Resistance % (95% CI)**					
	**Tilapia**			**Asian Seabass**		
	**Fish (*n*=157)**	**Water (*n*=45)**	**Total (*n*=202)**	**Fish(*n*=20)**	**Water(*n*=27)**	**Total (*n*=47)**
Ampicillin	30.6 (23.6-38.5)	57.8 (42.3-72)	36.6 (30.0-43.7)	45 (23.8-68.0)	22.2 (9.4-42.7)	31.9 (19.5-47.2)
Chloramphenicol	12.7 (8.1-19.2)	24.4 (13.4-39.8)	15.3 (10.8-21.2)	35 (16.3-59.1)	18.5 (7.0-38.7)	25.5 (14.4-40.6)
Ciprofloxacin	15.3 (10.2-22.1)	22.2 (11.7-37.5)	16.8 (12.1-22.8)	25 (9.6-49.6)	18.5 (7.0-38.7)	21.3 (11.2-36.1)
Colistin	21.7 (15.7-29.1)	6.7 (1.8-19.4)	18.3 (13.4-24.5)	35 (16.3-59.1)	3.7 (0.2-20.9)	17 (8.1-31.3)
Cefotaxime	0.6 (0.0-4.0)	0 (0.0-9.8)	0.5 (0-3.2)	0 (0.0-20.0)	0 (0.0-15.5)	0 (0.0-9.4)
Erythromycin	98.7 (95.0-99.8)	95.6 (83.7-99.2)	98 (94.6-99.4)	95 (73.1-99.7)	74.1 (53.4-88.1)	83 (68.7-91.9)
Gentamycin	5.1 (2.4-10.1)	2.2 (0.1-13.2)	4.5 (2.2-8.6)	0 (0.0-20.0)	0 (0.0-15.5)	0 (0.0-9.4)
Kanamycin	11.5 (7.2-17.8)	22.2 (11.7-37.5)	13.9 (9.6-19.6)	25 (9.6-49.6)	7.4 (1.3-25.7)	14.9 (6.7-28.9)
Nalidixic Acid	9.6 (5.7-15.6)	11.1 (4.2-24.8)	9.9 (6.3-15.1)	20 (6.6-44.3)	11.1 (2.9-30.3)	14.9 (6.7-28.9)
Streptomycin	16.6 (11.3-23.6)	31.1 (18.6-46.8)	19.8 (14.7-26.1)	10 (1.8-33.1)	0 (0.0-15.5)	4.3 (0.8-15.8)
Tetracycline	31.2 (24.2-39.2)	53.3 (38.0-68.0)	36.1 (29.6-43.2)	40 (20-63.6)	18.5 (7.0-38.7)	27.7 (16.1-42.9)
Ceftiofur	0 (0.0-3.0)	0 (0.0-9.8)	0 (0.0-2.3)	0 (0.0-20.0)	0 (0.0-15.5)	0 (0.0-9.4)
Trimethoprim	29.9 (23.0-37.8)	35.6 (22.3-51.3)	31.2 (25.0-38.1)	35 (16.3-59.1)	18.5 (7.0-38.7)	25.5 (14.4-40.6)

Table S2. Primers and PCR conditions for *Salmonella.*

Salmonella	**Target Genes**	**Primers**	**Sequence (5′–3′)**	**Product length (bp)**	**Ta (°C)**	**Reference**
Salmonella spp.	*invA*	Salm3	GCTGCGCGCGAACGGCGAAG	387	55	[108]
		Salm4	TCCCGCCAGAGTTCCCATT			
S. Enteritidis	*SelfA*	Sent F	AAATGTGTTTTATCTGATGCAAGAGG	299	55	[108]
		Sent R	GTTCGTTCTTCTGGTACTTACGATGAC			
S. Typhimurium	*fliC*	Styp F	CCCCGCTTACAGGTCGACTAC	433	55	[108]
		Styp R	AGCGGGTTTTCGGTGGTTGT			

Table S3. Primers and PCR conditions for *Vibrio.*

**Microbes**	**Target Genes**	**Primers**	**Sequence (5′-3′)**	**Product Length (bp)**	**Ta (°C)**	**Reference**
V. Parahaemolyticus	*toxR*	VP toxR 325F	TGTACTGTTGAACGCCTAA	503	55	[112]
		VP toxR 828R	CACGTTCTCATACGAGTG			
V. vulnificus	*vvhA*	vvhA 870F	ACTCAACTATCGTGCACG	366	55	[112]
		vvhA 1236R	ACACTGTTCGACTGTGAG			
V. cholerae	*toxR*	VC toxR 403F	GAAGCTGCTCATGACATC	275	55	[112]
		VC toxR 678R	AAGATCAGGGTGGTTATTC			

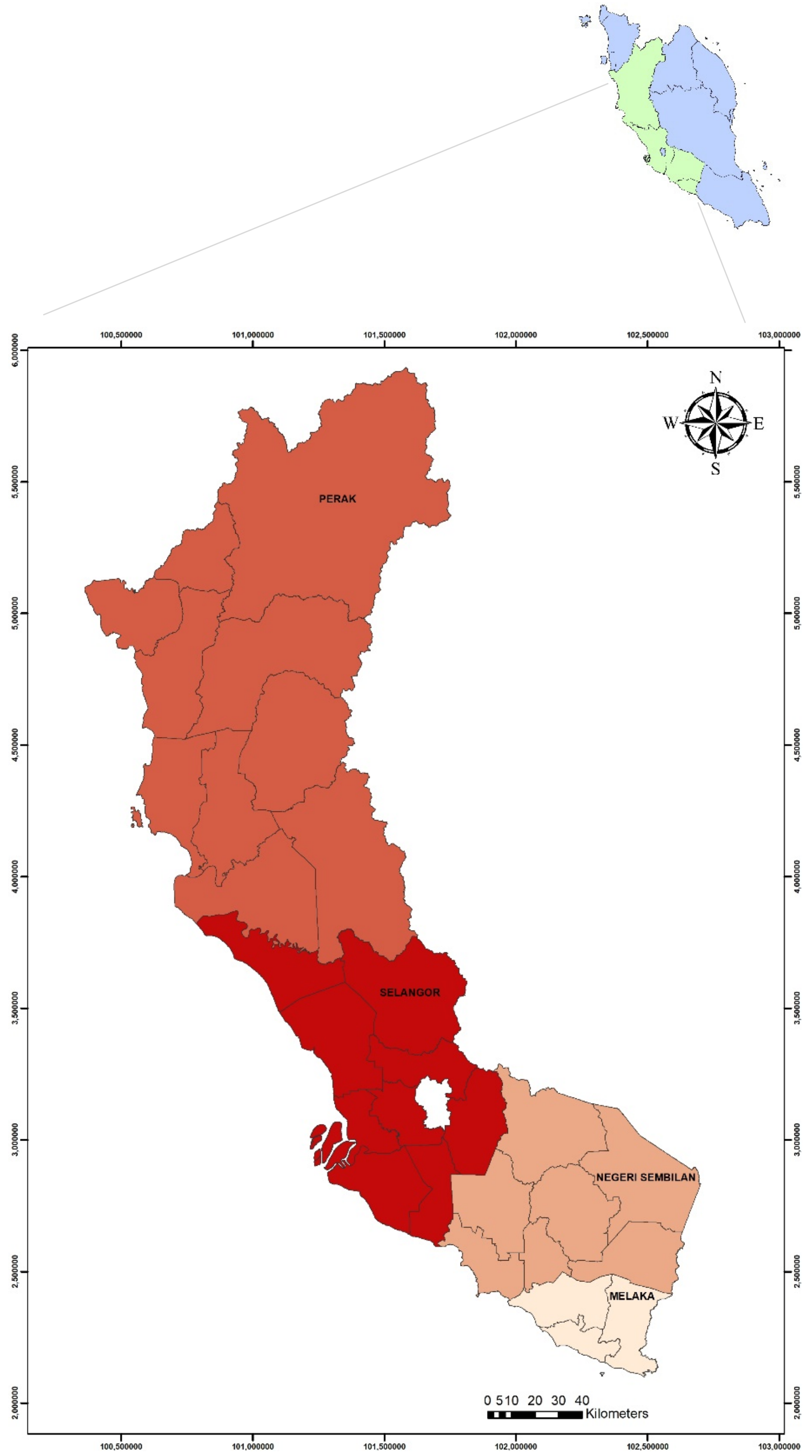

Figure S1. Map of study area indicating its four states in the west coast of peninsular Malaysia; Perak, Selangor, Negeri Sembilan and Melaka.
